# Pelvic floor muscle strength in primiparous women according to the delivery type: cross-sectional study**^1^**

**DOI:** 10.1590/1518-8345.0926.2758

**Published:** 2016-08-15

**Authors:** Edilaine de Paula Batista Mendes, Sonia Maria Junqueira Vasconcellos de Oliveira, Adriana de Souza Caroci, Adriana Amorim Francisco, Sheyla Guimaraes Oliveira, Renata Luana da Silva

**Affiliations:** 2 Master's Student, Escola de Enfermagem, Universidade de São Paulo, São Paulo, SP, Brazil.; 3 PhD, Associate Professor, Escola de Enfermagem, Universidade de São Paulo, São Paulo, SP, Brazil.; 4 PhD, Professor, Curso de Obstetrícia, Escola de Artes, Ciências e Humanidades da Universidade de São Paulo, São Paulo, SP , Brazil.; 5 Doctoral Student, Escola de Enfermagem, Universidade de São Paulo, São Paulo, SP, Brazil.; 6 Master's Student, Escola de Enfermagem, Universidade de São Paulo, São Paulo, SP, Brazil.; 7 MSc, Assistant Professor, Escola de Artes, Ciências e Humanidades, Universidade de São Paulo, São Paulo, SP, Brazil.

**Keywords:** Muscle Strength, Perineum, Postpartum Period, Obstetric Nursing

## Abstract

**Objectives:**

to compare the pelvic floor muscle strength in primiparous women after normal birth and cesarean section, related to the socio-demographic characteristics, nutritional status, dyspareunia, urinary incontinence, perineal exercise in pregnancy, perineal condition and weight of the newborn.

**Methods:**

this was a cross-sectional study conducted after 50 - 70 postpartum days, with 24 primiparous women who underwent cesarean delivery and 72 who had a normal birth. The 9301 PeritronTM was used for analysis of muscle strength. The mean muscle strength was compared between the groups by two-way analysis of variance.

**Results:**

the pelvic floor muscle strength was 24.0 cmH2O (±16.2) and 25.4 cmH2O (±14.7) in postpartum primiparous women after normal birth and cesarean section, respectively, with no significant difference. The muscular strength was greater in postpartum women with ≥ 12 years of study (42.0 ±26.3 versus 14.6 ±7.7 cmH2O; p= 0.036) and in those who performed perineal exercises (42.6±25.4 11.8±4.9 vs. cmH2O; p = 0.010), compared to caesarean. There was no difference in muscle strength according to delivery type regarding nutritional status, dyspareunia, urinary incontinence, perineal condition or newborn weight.

**Conclusion:**

pelvic floor muscle strength does not differ between primiparous women based on the type of delivery. Postpartum women with normal births, with higher education who performed perineal exercise during pregnancy showed greater muscle strength.

## Introduction

The pelvic floor muscles play an important role in supporting the pelvic and abdominal organs and controlling urinary and fecal continence, in addition to their role in the sexual function^1^. Pregnancy and childbirth, however, influence this musculature and can decrease its tone, leading to a set of problems known as pelvic floor dysfunction (PFD), such as urinary incontinence (UI) and dyspareunia, and other conditions that may be transient or permanent^2^ ^-^ ^3^. These issues can negatively impact the sexual, physical and professional activities of women.

Urinary incontinence is the involuntary loss of urine, with negative impacts on women in terms of their quality of life, and is considered a social and hygiene problem^4^. In a systematic review, the combined prevalence of any postpartum UI was 33% in all women; factors such as parity, delivery type, and exercise seem to influence the occurrence of this problem, however the studies are still controversial^2^. The average prevalence of UI in multiparous women was greater than in primiparous women (36.6% vs. 28.7%). In addition, the prevalence of UI in the instrumental postpartum was approximately twice compared to cesarean section (32% vs 15%)^2^. Another study found a positive correlation between normal delivery and UI, but not for cesarean section, which was assigned as a protective factor for UI. After aanalyzing the relationship between UI and the number of pregnancies and normal deliveries, the authors concluded that the greater the number of these two variables, the greater the rate of UI, showing that pregnancy and normal delivery are also risk factors for UI^5^. 

A cohort conducted in Sweden with 5,236 women found that vaginal birth, compared to cesarean section, increased the risk of UI by 275% for the period of ten years after childbirth, and in 67%, for 20 years after delivery. However, the authors concluded that eight to nine women having cesarean sections would be necessary to avoid one case of UI, using the amount needed to be treated^6^. On the other hand, in another study no significant difference was identified between the prevalence of UI two years after vaginal childbirth, compared to cesarean section^7^.

Scientific evidence shows that exercise for pelvic strengthening must be used and if performed early in primiparous women can prevent UI at the end of the pregnancy and postpartum^8^.

A cohort study performed in Australia with 1,244 nulliparous found dyspareunia in 24% of them after 18 months following childbirth. Obstetric interventions such as vaginal birth with vacuum extraction and emergency or elective cesarean section were associated with the persistence of this symptom in that population (OR=2.28, p 0.005; OR=2.41, p 0.001; OR=1.71, p=0.087, respectively)^9^. Another cohort study was conducted with women who reported having dyspareunia or not having it. No significant difference was identified between the groups in relation to vaginal pressure at rest, perineal muscle strength and vaginal endurance, six and 12 months after delivery. Also, no significant difference was identified between the groups according to the delivery type, episiotomy, third and fourth degree perineal lacerations, or weight of the newborn^10^. 

The assessment of pelvic floor muscle strength (PFMS) is important for the prevention, diagnosis and treatment of the pelvic floor dysfunction (PFD). The PFMS can be assessed at rest or during activity, by means of resistance and muscular contraction during the gynecological examination, using methods such as: vaginal digital palpation, perineometry, ultrasonography, electromyography, manometry, and vaginal cones^11^

In a study that tested the reliability of the Peritron^TM^, resistance and PFMS was verified by means of two measures on the same day, with one hour between them, in order to assess intra-day reliability. After five days, a third measure was conducted to check the between-days reliability. The authors found a high level of agreement between the measurements. Thus, the Peritron^TM^ can be considered a reliable method for measuring the resistance and PFMS^11^. 

Thus, the objectives of this study were: to analyze PFMS in postpartum primiparous women following normal birth and cesarean section; compare the PFMS of normal birth and cesarean section postpartum women relating to socio-demographic characteristics, nutritional status, UI, dyspareunia, exercise during pregnancy, perineal condition, and weight of the newborn.

## Method

This was a cross-sectional study on PFMS in primiparous women, 50 to 70 days after childbirth, according to delivery type, conducted in a maternity hospital and Basic Health Unit - Unidade Básica de Saúde (UBS) - of the municipality of Itapecerica da Serra, São Paulo, Brazil.

The sample was composed of women who met the following inclusion criteria: having only one normal birth or caesarean section of a full term newborn (37 to 42 weeks), single and alive with cephalic presentation at birth; who did *not undergo* abdominal or urogenital surgery; without diseases or physical conditions that could interfere with PFMS (pelvic or spinal injury, diabetes, pelvic organ prolapse, neurological disorders); without problems of communication due to hearing limitation or speech acuity. The exclusion criteria were: difficulty in inserting the perineometer into the vagina and complications in the healing process of the perineal region due to local trauma. The sample size was calculated based on the averages of PFMS in women with normal and cesarean section delivery of a previous study^13^. When comparing this data, the *Cohen's d effect size* of 0.669 was found; assuming a type I error of 5% and 90% test power, 96 mothers were required.

The data of births registered in the hospital maternity area, during the 2011-2012 period, showed that for each woman having a caesarean section, three childbirths occurred. Considering this distribution, 24 post-caesarean women and 72 normal birth postpartum women were required. The study was approved by the Research Ethics Committee of the school of nursing, University of São Paulo (CAAE: 13545113.5.0000.5392) and women's participation was voluntary, after receiving orientation and signing the Terms of Free and Informed Consent. As for the device used in this study, it is important to clarify that there is no link between the researchers and the manufacturer. The data were collected in two steps, between January and September of 2014. The data collection form was adapted from a study which also assessed PFMS(14), and was tested prior beginning the data collection. Two researchers previously prepared on the proper use of the perineometer performed the data collection. 

*Step 1* was conducted in the hospital maternity area and consisted of recruitment, interview and data collection from the medical record. Thus, a return visit was scheduled after 50 and 70 days from delivery to the original UBS or the maternity unit where they were recruited. Telephone contact was made between one and two days before the appointment in order to confirm the presence of a woman who recently gave birth. In case of a no-show, the appointment was scheduled again by phone call. 

Step 2 consisted of the second part of the interview and the assessment of PFMS. An electronic pressure meter, the PeritronTM, model PRN09301 (Laborie, Canada), was used to measure the PFMS, which consists of a silicone vaginal probe of 8 cm long and 3 cm in diameter which registers muscle contraction using a portable microprocessor, numerically, from 0.1 centimeters of water (cmH_2_O). The unit did not differentiate pelvic muscles or abdomen contractions. During the evaluation of the PFMS, the movement of accessory muscles and the Valsalva maneuver were controlled by means of visual observation. The procedure of PFMS measurement by perineometry was conducted by one of the researchers and followed the methodology described in a published study^15^. The data registered were double entered in the Statistical Package for the Social Sciences (SPSS), version 22.0 for Mac. The statistical analysis was performed by validation of the database and importing of the data into the Excel application. The analysis of variance (ANOVA) was used to compare the mean of PFMS between women who had normal birth and by cesarean section. The *two* -*way* analysis of variance ANOVA was used to assess the relationship of sociodemographic characteristics, nutritional status, UI, dyspareunia, perineal exercise and weight of the newborn. For association of PFMS with delivery and perineal condition data, the *one* -way ANOVA was applied. The significance level of 5% was adopted for all tests.

## Results

Among the 236 eligible primiparous women, 51 refused to participate in the study because they lived in another municipality, 15 were not addressed due to early hospital discharge, and one was not included because she underwent episiotomy and cesarean section, as well. Therefore, 169 women were recruited, and of these, 73 did not attend the study sessions, and two were considered as a loss. Thus, the final sample consisted of 96 primiparous women, who attended the two steps of the study. Due to the high percentage of losses (43.2%), a comparative analysis between the losses and the final sample was performed, showing that the losses were random and did not influence the sample.

The mean age of participants was 21.7 (± 4.8) years old, minimum of 13 years and maximum of 37; 36.5% were up to 19 years. Most women reported being mixed race (56.3%), having between nine and 11 years of education, living with a partner (78.1%), and being without remunerated employment (61.5%).

The mean PFMS among women with normal delivery was 24 (dp = 16.2) cmH_2_O , with those who had a cesarean section at 25.4 (SD = 14.7). Although the PFMS after cesarean section was 1.4 cmH_2_O higher than with normal delivery, the difference was not significant according to the type of delivery (p = 0.697). The mean values of the PFMS in relation to socio-demographic variables showed significant difference only when the woman had 12 years or more of education (Table 1). In the categories of 8 and 9 to 11 years of education, this difference was not significant, showing a significant reversal, represented in the Figure 2. In terms of urinary conditions, smaller values of PFMS were found after normal birth, without significance, as demonstrated by Table 1.

**Figure 1:**
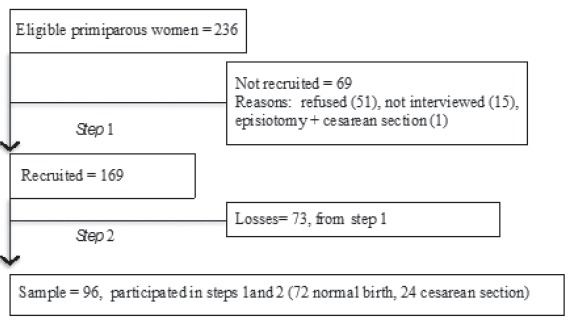
Flowchart of the research participants. Itapecerica da Serra, SP, Brazil, 2014

**Figure 2:**
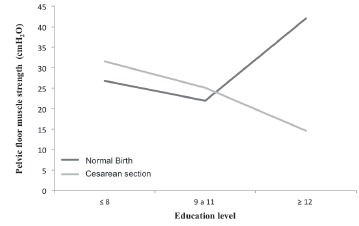
Interaction between education level and the type of delivery on pelvic floor muscle strength (PFMS). Itapecerica da Serra, SP, Brazil, 2014

**Table 1:** Mean pelvic floor muscle strength according to childbirth type, sociodemographic characteristics, nutritional status, urinary incontinence, dyspareunia, perineal exercise, perineal condition and weight of the newborn. Itapecerica da Serra, SP, Brazil, 2014

Variable		PFMS ^*^ (cmH_2_O)					p-valor
		Normal birth			Cesarian section		
		n	Mean (SD^†)^		n	Mean (SD)	
Age (years)							0.390^‡^
	≤ 19	28	26.0 (14.8)		7	21.2 (12.9)	
	20 - 24	28	26.4 (18.3)		9	30.4 (16.1)	
	25 - 29	11	21.1 (12.0)		6	23.0 (17.3)	
	≥ 30	5	5.5 (8.4)		2	25.2 (3.1)	
Skin collor							0.406^‡^
	Mixed	40	25.5 (15.9)		14	26.5 (15.2)	
	White	23	20.1 (15.6)		7	25.8 (16.4)	
	Black	8	30.6 (17.4)		3	19.3 (10.4)	
	Yellow	1	0.0 -		-	-	
Education (years)							0.036^‡^
	≤ 8	10	26.8 (20.7)		6	31.5 (10.8)	
	9 - 11	57	21.9 (13.4)		15	25.1 (16.2)	
	≥ 12	5	42.0 (26.3)		3	14.6(7.7)	
Marital status							0.339^‡^
	Living with partner	55	23.1 (16.9)		20	26.8 (14.2)	
	Does not live with partner	10	25.9 (14.0)		1	6.7 -	
	No partner	7	27.7 (14.9)		3	22.3 (19.3)	
Remunerated employment							0.767^‡^
	Yes	41	24.9 (14.8)		18	24.8 (11.5)	
	No	31	23.3 (17.4)		6	25.6 (15.9)	
Nutritional status							0.584^‡^
	Low weight	5	23.4 (18.1)		0	-	
	Adequate	43	23.9 (14.8)		12	20.5 (10.0)	
	Overweight	20	23.1 (15.4)		7	25.6 (20.0)	
	Obesity	4	29.1 (34.6)		5	36.9 (11.6)	
ICU during pregnancy							0.296^‡^
	Yes	67	19.9 (20.1)		14	27.0 (14.9)	
	No	29	25.4 (14.5)		10	24.3 (15.0)	
ICU after delivery							0.894^‡^
	Yes	59	22.4 (20.8)		20	25.0 (14.4)	
	No	13	24.3 (15.2)		4	25.5 (15.1)	
UI that persists for two months postpartum (n=17)							0.448^‡^
	Yes	6	12.2 (9.9)		3	21.9 -	
	No	7	34.4 (24.5)		1	26.0 (17.5)	
Dyspareunia (n=77)							0.361^‡^
	Yes	30	24.3 (17.3)		10	24.8 (14.6)	
	No	28	21.5 (13.1)		9	29.3 (14.6)	
Perineal exercise during pregnancy							0.010^‡^
	Yes		42.6 (25.4)		2	11.8 (4.9)	
	No		22.6 (14.7)		22	26.7 (14.7)	
Perineal condition							0.677^§^
	Intact	11	23.5 (16.2)				
	First degree laceration	22	27.5(14.7)				
	Second degree laceration	11	21.0 (13.5)				
	Third degree laceration	1	8.8 -				
	Episiotomy	27	23.0 (15.2)				
Weight of newborn							0.732^‡^
	≤ 3,500	57	23.7 (16.4)		14	26.2 (14.7)	
	> 3,500	15	24.8 (16.0)		10	24.3 (15.5)	

* Pelvic floor muscle strength

†Standard deviation ‡ Two-way ANOVA § ANOVA

|| Incontinence

**Figure 3:**
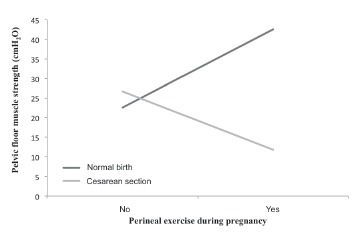
Interaction between the perineal exercise in pregnancy and delivery type on pelvic floor muscle strength (PFMS). Itapecerica da Serra, SÃO PAULO, Brazil, 2014

Comparing the PFMS mean with perineal exercise during pregnancy, interaction was observed between practicing perineal exercise and the type of childbirth (p = 0.010). Primiparous women who exercised the perineum during pregnancy, and who had a normal birth, showed significantly higher values of PFMS compared to women who experienced a cesarean section, as shown in Figure 3.

In relation to the perineal condition, the PFMS among primiparous women who had either an intact perineum or an episiotomy were similar. No significant differences were found between the means on PFMS and the weight of the newborn according to the type of childbirth, described in Table 1.

## Discussion

This investigation sought to compare the PFMS according to the type of childbirth, associating the sociodemographic characteristics and delivery, to PFD and perineal exercise in pregnancy. This data contributes to the profile of the PFMS and provides indicators that could support the option of choice for women regarding the type of delivery.

Normal birth is seen as a factor that promotes a weakening of the pelvic floor muscles. Thus, the choice of the woman for caesarean section is often associated with the prevention of morbidities related to the lost of PFMS. However, the current study did not identify an influence on type of childbirth and PFMS between 50 and 70 days after the childbirth. This result differs from that found by a study^16^ that compared nulliparous women with primiparous women after normal and cesarean section delivery, in which a decline in FMAP was identified among primiparous women with normal birth, compared to those with a cesarean section. 

The normal birth increased the risk of decrease the PFMS by 2.58 and 2.31 times after four and six months postpartum, respectively, while for the cesarean section women the risk was 1.56 and 1.37 times (at four and six months, respectively). This divergence of results can be justified by the use of different devices to measure the PFMS and the variation in the time at which the evaluation was performed.

The great variability in the assessment periods for PFMS complicates the comparison between the results of this study with those of previous surveys, as the literature suggests that the mean PFMS varies during the puerperal period, with a tendency to increase over time, independent of the type of birth^17^. This variation can be observed in surveys conducted with primiparous women at 45 postpartum days, which identified PFMS of 8.3 cmH_2_O and 13.7 cmH_2_O (postpartum normal birth and cesarean section, respectively)^18^ ^)^ and higher measures at 98 days and 12 months postpartum, 54.1 cmH_2_O and 59.9 cmH_2_O, respectively^17^. That variation can be observed even in studies that used devices with different units of measurement ^(^ ^13^ ^-^ ^14^. 

Considering the socio-demographic characteristics in this study, as well as in others^14^ ^-^ ^15^, maternal age, marital status, skin color and remunerated employment did not influence PFMS. Although some authors suggest that age and skin color are related to lower PFMS after childbirth^19^, in the current survey, a significant difference in PFMS was identified only in relation to education level. Primiparous women over 12 years of education and normal birth showed a significantly higher PFMS value. However, these data should be viewed with caution, as the sample size was small in that category.

The literature did not present studies that investigated the relationship of PFMS with education. However, the authors suggest that the level of education may be associated with other factors such as socioeconomic condition, i.e. higher education, in general, is related to increased socioeconomic condition which in turn allows access to better nutrition, physical activity, and greater access to health care among others^19^. However, the socio-economic condition was not addressed in the current research, preventing more comparisons. 

As for the nutritional status, although there is no parameters for the body mass index during the puerperal period, according to the Atalah classification(20) for the gestational period, most women in this study presented adequate nutritional status at the time of evaluation of the PFMS, and no significant differences were found in PFMS in relation to nutritional status according to the type of delivery. However, one study(18) that compared the PFMS of nulliparous and primiparous women suggested that being overweight influenced the results.

In this investigation, UI showed higher prevalence in pregnancy compared to postpartum, and most women complaining about UI at two months postpartum had urine loss during the pregnancy. Although gravidic changes may contribute to UI after childbirth^21^, some authors present evidence that, when present in pregnancy, this condition becomes a risk factor for its occurrence also in postpartum^7^ ^,^ ^21^ ^-^ ^22^_._

Despite the fact that results showed lower values of PFMS in women with UI who were postpartum after normal compared to cesarean section delivery, this difference was not significant. The result of a prospective study^22^ is similar to the findings of the present investigation. The authors concluded that PFMS did not interfere with the symptoms of UI, regardless of the type of delivery. 

Few recent mothers reported doing some kind of pelvic strengthening exercise during pregnancy and after childbirth, with the most cited being Kegel exercises and holding the ureterovesical jet. Those who had normal childbirth and exercised their perineum during pregnancy showed significantly higher values of PFMS compared to post-caesarean women, however it should be noted that it was not asked if the exercise was performed on a regular basis or if the women received guidance on how to do it. However, it is possible to affirm that perineal exercise can bring benefits to those who do it. Results that reinforce this assertion were demonstrated by studies that evaluated the exercises both in pregnancy as well as in the puerperium, indicating that strengthening of the pelvic muscles contributes to the improvement of the symptoms of UI^8^ and sexual function^23^. Thus, the inclusion of an exercise program for prenatal care could have beneficial effects for the strengthening of this musculature and prevention of UI.

In this study, most primiparous women returned to sexual activity between 50 and 70 days after delivery and about half of them mentioned dyspareunia, however, as with other studies^10^ ^,^ ^15^, no difference was found in PFMS related to the type of childbirth regarding this complaint.

As in a prior research study^13^, PFMS was not associated with the perineal condition during normal birth. But, this variable seems to be related to sexual dysfunction. A prospective type study, aiming to identify the extent of sexual dysfunction with three months postpartum of normal delivery, found an association between perineal injury and increased rate of dyspareunia and reduction in levels of libido, orgasm and sexual satisfaction^24^. Thus, it is important to prevent perineal trauma, because even if no influence of this variable on PFMS is identified, it can be a determinant for the resumption of sexual function after birth. The PFMS was not associated with the weight of the newborn, regardless of the type of delivery. Studies evaluating the relationship between these two variables were not found. However, the literature shows that fetal weight greater than 4 kg is a predictor for urinary and fecal incontinence, suggesting that to prevent such problems, normal birth of macrosomic babies should be avoided^25^.

The lack of standardized methods for assessing PFMS within research hinders the comparison of the values of this variable. In this sense, the current study used a method previously employed in most current studies^15^ ^,^ ^26^ in an attempt to standardize such measures and use it as a basis for other studies, facilitating the comparison of results, and assisting in the evaluation of pelvic muscle tone after childbirth. 

Our results also provide support so that the professional can guide pregnant women about the factors that interfere with PFMS, providing one more element to support the decision-making of the woman related to the type of delivery. Furthermore, the results indicate that special attention should be devoted especially to pregnant women with lower education levels and with UI during pregnancy, in order to improve perineal care. In this way, the direction to practice, or the inclusion of an exercise program to strengthen the muscles of the pelvic floor, in prenatal care, can have positive impact on PFMS after normal birth. 

## Limitations

A follow-up loss of about 40% related to the non-return of women to puerperal consultation was found in this study. Thus, these participants were replaced and the sample reached, at the end of the survey, the number previously established in the calculation of the sample. In addition, the comparative analysis between the losses and the participants in the study sample showed that they differed in only three days on gestational age. That loss can be attributed to geographical distribution between the basic health units and the residence of the participants.

Another limitation of this study could be related to the single evaluation of PFMS after childbirth, which precluded a determination of whether there are long-term influences of the type of childbirth on this type variable.

## Conclusion

No difference was found in the PFMS of postpartum mothers who had normal and cesarean deliveries. Postpartum primiparous women with 12 or more years of education presented with a higher PFMS. The perineal exercises in pregnancy were associated with increased PFMS in postpartum mothers who had a normal delivery.
